# Setting-up nurse-led pilot clinics for the management of non-communicable diseases at primary health care level in resource-limited settings of Africa

**Published:** 2009-10-24

**Authors:** Andre Pascal Kengne, Eugene Sobngwi, Leopold Fezeu, Paschal Kum Awah, Sylvestre Dongmo, Jean-Claude Mbanya, The CENHIP Investigators

**Affiliations:** 1 Health of Population in Transition (HoPiT) research group, Yaoundé, Cameroon,; 2 The George Institute for International Health, The University of Sydney, Australia,; 3 Institute of Health and Society, Medical School, Newcastle University,; 4 Epidemiologic and Biostatistics Research Unit, Inserm Unit 780, Villejuif, France,; 5 University of Paris XI, Kremlin Bicêtre, France,; 6 Department of Anthropology, Faculty of Arts, Letters and Social Sciences, University of Yaoundé I, Cameroon,; 7 Bafut district hospital, Cameroon,; 8 Department of Internal Medicine and Specialties, Faculty of Medicine and Biomedical Sciences, University of Yaoundé 1, Cameroon.

**Keywords:** chronic diseases, nurse-led clinics, diabetes, hypertension, epilepsy, asthma, primary health care, Cameroon, sub-Saharan Africa

## Abstract

**Background:**

This article describes the setting-up process for nurse-led pilot clinics for the management of four chronic diseases: asthma, type 2 diabetes mellitus, epilepsy and hypertension at the primary health care level in urban and rural Cameroon.

**Methods:**

The Biyem-Assi urban and the Bafut rural health districts in Cameroon served as settings for this study. International and local guidelines were identified and adapted to the country’s circumstances. Training and follow-up tools were developed and nurses trained by experienced physicians in the management of the four conditions. Basic diagnostic and follow-up materials were provided and relevant essential drugs made available.

**Results:**

Forty six nurses attended six training courses. By the second year of activity, three and four clinics were operational in the urban and the rural areas respectively. By then, 925 patients had been registered in the clinics. This represented a 68.5% increase from the first year. While the rural clinics relied mainly on essential drugs for their prescriptions, a prescription pattern combining generic and proprietary drugs was observed in the urban clinics.

**Conclusion:**

In the quest for cost-effective health care for NCD in sub-Saharan Africa, rethinking health workforce and service delivery has relevance. Nurse-led clinics, algorithm driven service delivery stands as alternatives to overcome the shortage of trained physicians and other issues relating to access to care.

## Background

The most important challenges to good quality health care in developing countries include the changing pattern of disease occurrence and shortage of adequately trained health personnel are among the most important. The changing pattern of disease occurrence is characterized by the re-emergence of some infectious diseases, but also by the rapid emergence of chronic non-communicable diseases (NCD) and related risk factors [1]. Health systems in sub-Saharan Africa, geared so far toward the control of infectious diseases are less prepared to face the emerging challenges resulting from NCDs. As a result, NCD are often ignored, under-diagnosed with a high burden of acute and chronic complications. While health systems in these countries are being realigned to integrate aspects of NCD care, an issue of major importance to be addressed urgently relates to the lack of adequately trained health workforce for the management of these conditions. Many reasons account for the chronic lack of trained health personnel. These include the lack of training facilities and resources, and the high migration rate among those available in quest for better working and living conditions in more developed settings [2]. With a health worker-to-population ratio 0.8 per 10,000 inhabitants, there are all indications that developing countries of sub-Sahara Africa (SSA) will not cope with the high demand of caring for chronic diseases, in addition to the still prevalent infectious diseases [3]. More importantly, the few available medical doctors are predominantly based in major urban cities and therefore pose additional problems of access to care for population residing in rear and rural areas.

The overall goal of the Essential NCD health intervention project (ENHIP) was to contribute to strategies for the effective control and prevention of common NCDs within SSA [4]. The program in Cameroon was implemented in the capital city (Yaoundé) and in the rural health district of Bafut. As part of the ENHIP, a protocol driven nurse-led care was set-up for the management of asthma, diabetes mellitus, epilepsy and hypertension at primary health care levels. This paper describes the setting-up process for nurse-led clinics for the management of the four above-mentioned chronic conditions. Reasons for selecting these four conditions have been described in details elsewhere [4].

## Methods

### Study settings

Cameroon is a middle-income Central African country of 475000 km ^2^ an overall 16 million population (45% urban). Life expectancy at birth in Cameroon is 55 years and lifestyle varies from traditional hunter-gathering activities with moderate to intense energy expenditure in rural communities to typically westernized lifestyle in major cities. The country has a public health service based on 10 administrative regions, with a network of nearly 268 hospitals and 1000 health centres. Bafut (rural) and Biyem-Assi (urban) health districts were used for this study. Bafut is a rural sub-division in the highlands of North West Cameroon with an estimation 100000 population living mainly on subsistence farming. Biyem-Assi is a health district of 173393 population in the capital city (Yaoundé). The population of this district is composed mainly of civil servants, businessmen and students.

### Origin of patients followed up in the clinics: Prevalence and risk factors survey

**Figure 1:**
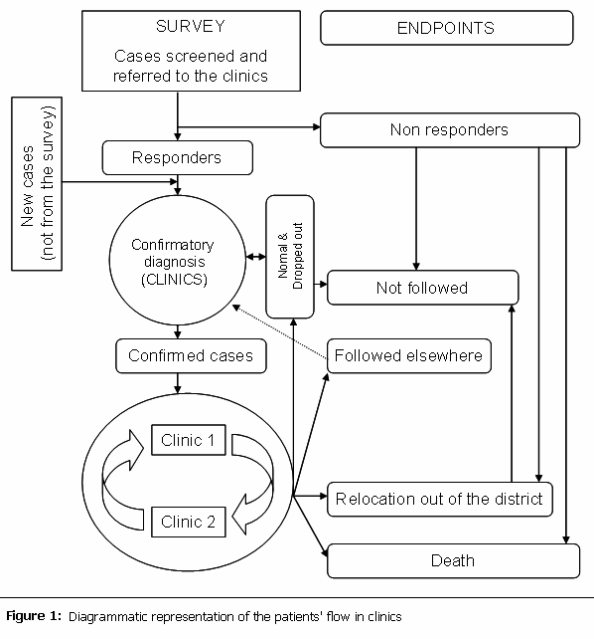
Diagrammatic representation of the patients' flow in clinics

Between November 1997 and February 1998, a population-based epidemiological survey was conducted in two health areas of Bafut (Manji and Nsem health areas with a total population of 17000), and one heath area of Biyem-Assi (Biyem-Assi health area, total population 15645). The aim of this survey was to determine the prevalence of the four chronic diseases under consideration and their related risk factors, to identify patients eligible for follow-up in the clinics ( Figure 1 ) and to sensitize populations on the growing importance of chronic diseases. This program received approval from the National Ethical Committee and was authorized by the Ministry of heath. In addition, local administrative, traditional, health authorities and key leaders were identified in each district and invited to provide their community support to this initiative.

### Development of training tools and patients follow-up materials Guidelines

One separate guideline was developed for each condition. These were subsequently merged into a single training package. Each guideline has themes relating to the definition of the disease, diagnosis and treatment under regular and specific conditions, patient education, follow-up, referral and review criteria. The Epilepsy guideline was based largely on the ‘ICBERG’ publication, “The management of epilepsy in developing countries” [5]. Asthma guideline was adapted from two sources. Firstly, the evidence based guideline for the primary care management of asthma in adults developed at the Centre for Health Services Research University of Newcastle upon Tyne [6] and secondly the guidelines developed by the British Thoracic Society [7]. It was intended for children above 5 years and adults. The diabetes guideline was largely based on the guidelines for the management of diabetes in Africa [8]. Other guidelines and protocols were also consulted. The diabetes ‘audit protocol’ from the Eli Lilly National Clinical Audit Centre [9] was useful in suggesting the choice of review criteria. The diagnostic criteria for diabetes were in line with the recommendations from a WHO consultative committee [10]. The hypertension guideline was adapted from the British Hypertension Society guidelines [11] and the related publication “ABC of hypertension” [12]. The instructions for measuring blood pressure were based on recommendations from British Hypertension Society [13].

### Patient forms

A generic combined initial form was developed to screen new patients for the presence of each of the four conditions. Thereafter, follow-up forms were specific for each condition. Duplicate files were allowed for patients cumulating two conditions. A tracking form was introduced to trace patients lost to follow-up.

### Registers and diaries

In addition to the forms, each clinic has registers for summarizing data for each patient per visit. There was one register per clinic for each condition. These registers served as backup method, but also as tools for validating records in patients’ files. A diary was also available to organize the visit appointment and trace patients lost to follow-up.

### Patient’s self-held note book and patient education material

Each patient was encouraged to acquire a self-held note book in which a summary of consultations and prescriptions was kept at each visit. In addition, a range of leaflets were available to backup education on diseases and risk factors.

### Training and retraining

An initial training course was organized in each district prior to the baseline risk factors and diseases survey. Follow-up trainings were thereafter organized as needed. This training was design to standardize clinical practice among nurses in charge of the clinics. In addition to nurses directly in charge of the clinics, training was also provided to most nurses in the health district to improve diagnosis and referral of cases to the pilot clinics and provide for the extension of the activities to the entire health district when the program become effective.

Initially designed on a typical medical school model, the lectures were progressively modified according to the feedback from the trainees. This feedback was obtained by three different ways: pre and post -course evaluation, reports from trainees and rapid evaluation of the quality of care delivered in the pilot clinics by the trainees. Basically, the course aimed at achieving good training of primary health care workers in the recognition of clinical presentation and risk factors of diabetes, hypertension, asthma and epilepsy; accurate diagnosis of the above; education of patients with those diseases; principles of management of those NCDs; record keeping and referral scheme. Training methods included: pre-course assessment; formal lectures; demonstration; discussions; practical sessions and post-training evaluation. Course facilitators were medical doctors (usually 2–3 per course) with experience in the field of these NCDs, assisted in the follow-up courses by nurses in charge of the clinics. Courses were organized in one health facility of the district and each course ran for three consecutive days in rural areas and six half-days in the urban site. Certificates of attendance were delivered to participants at the end of the training course.

### Setting up of pilot clinics

In addition to the instruments described above, each clinic was supplied with basic material for diagnosis and follow-up of the four conditions. These included mercury sphygmomanometer, weight and high scales, tapes, blood glucose monitors and relevant test strips, pick-flow-meters, other consumables like finger pricking device and needles, cotton, spirits, disposable gloves. In addition, health facilities were encouraged to acquire drugs for those conditions available in the essential drug list. Throughout the duration of the program, all clinics were supplied with glucose test strips free of charge. To ensure the sustainability of the program, patients were encouraged to pay for their medications. In the rural site, antiepileptic medications were provided free of charge to all patients. The first cohort of patients registered in clinics was referred from the population-based survey. Other patients were attracted as the activities of the clinic became popular in each site ( Figure 1 ). The program started with two pilot clinics in each heath district. The frequency of clinic activities was independently defined by each health facility as a function of the workload, staff availability, with provision for resources to assure concomitant smooth running of other activities in the health facility.

### Incentives

To encourage nurses and assist health facilities to cope with any additional workload resulting from the establishment of NCD clinics, monthly fees were paid to selected hospital on a flat rate base. This money was to be used both for nurses in charge of the clinics, but also for their colleagues who seconded them in all other hospital duties during NCD clinic hours.

### Review process and review criteria

The activities of the nurses were closely monitored by physicians at the beginning of the process. However, as they became more confident they were able to run the program with less monitoring. At the district level, once every two weeks, the district medical officer reviewed the activity of each clinic. In addition, once a month the central coordinating team reviewed all clinics. At these review visits, gaps in the practice were discussed with nurses in charge of the clinics. A set of review criteria and quality standards were defined to monitor the implementation of guidelines within each health facility. These review criteria were ‘must do’ criteria and the quality standards were deliberately set at minimum desired levels. These included: screening/detection and recording of patients on registers; adherence to diagnostic criteria; appropriateness of initial assessment and investigation; appropriateness of treatment and follow up; correct management, according to the guideline, in the event of an acute deterioration; assessment of patient compliance with treatment; outcomes; equipment and drug supply. The intervention went on for two consecutive years. A comprehensive review was performed at the end of each year. Outcomes specific to each of the four conditions are available in more details elsewhere [14–17].

## Results

### Nurses trained and clinics set-up

By the end of the intervention, six nurse-led clinics were operational in the two health districts. While two clinics were set-up in each district at the beginning the project, the 3 ^rd^ clinic was opened at the start of the second year in the urban, and the end of the same year in the rural district. One clinic in each district was hosted by a public health facility while the two others were located in private hospitals. In all, six training and follow-up courses were organized, three in each district, with a total of 18 nurses trained in the urban setting against 28 in the rural district. By the end of the program, all nurses in the vicinities of the rural district have attended at least one of the training courses.

### Clinic forms

Eleven forms were developed and updated yearly. A generic screening form was used to assess each patient at his first presentation in the clinic for asthma, diabetes, epilepsy or hypertension. In addition to classical clinical features of each condition, this form also assessed for the presence of risk factors, and the history of the disease in those previously diagnosed. For asthma and diabetes, a complementary initial assessment form was available for specific information not captured by the main initial form. For each of the four diseases, there was a follow-up form used at each clinic visit to monitor symptoms and follow-up parameters, compliance to treatment and side-effects and the presence of complications. With the exception of asthma, all follow-up forms were designed in such a way that each page should cover three consecutive visits. Such design has the advantage of providing for direct comparison of follow-up information across visits for each patient. The annual review form for diabetes and hypertension was used to evaluate changes in lifestyle and the presence of chronic complications. Finally, a defaulter questionnaire was available to trace participants lost to follow-up. The final versions of these forms are available in additional material.

### Patients flow in the clinics

At the first annual evaluation, 549 patients (64.5% rural) were registered in urban and rural clinics. Hypertension was the leading condition with 274 patients (58% rural), followed by epilepsy (146 patients, 92.5% rural), diabetes mellitus (66 patients, 42.4% rural) and asthma (63 patients, 50.8% rural). At the second year’s evaluation, in all 925 patients were registered in the clinics. This represented a 68.5% increase from the first year evaluation. While in the rural site the number of patients for all four conditions increased between the two evaluations, this number remained stable for epilepsy and asthma in the urban site. The maximum percentage change was observed for diabetes in the urban area (373.7%) and asthma in the rural area (171.9%). In general, rural clinics attracted more patients than the urban ones ( Table 1 ).

### Treatment modalities

Table 2 summarizes the drug prescriptions in the clinics at the second year’s evaluation. Urban patients with asthma or epilepsy, although registered with the clinics continued to receive care from the attending healthcare provider prior to implementing this program. Their treatment therefore was out of control of nurses in charge of the clinics and is not reported here.

While rural clinics relied mainly on essential drugs for their prescriptions, a prescription pattern combining generic and proprietary drugs was observed in the urban clinics. In the rural clinics, no patient with asthma or epilepsy was on combined medications; otherwise aminophilline and Phenobarbital were drugs more prescribed for asthma and epilepsy respectively. In patients with hypertension alone, 8.7% to 22.4% were on lifestyle only; 10.1–39.7% were on drug combination including at least a thiazide diuretic, and the remaining were on monotherapy (rural patients) or other drugs associations (urban patients). Blood pressure lowering treatment among those with diabetes in rural area was dominated by thiazide diuretics, found in at least 66.6% of prescriptions, either alone or in combination.

Among their urban counterparts, although thiazide diuretics were still highly prescribed, many other blood pressure lowering agents were also found. Less than 10% of patients with diabetes were on lifestyle measures alone to control their blood glucose. Sulphonamides were the most prescribed oral anti-diabetic agents, followed by metformin either alone or in association. Blood glucose lowering treatment pattern did not differed that much between diabetic patients with hypertension and those without.

## Discussion

The implementation of this program was successful as evidenced by the number of nurses trained, the wide range of clinic tools developed, the number of clinics set-up in each health district and the patients flow in pilot clinics. This success was more pronounced in the rural site and supports the view that nurse-led clinics are good alternatives for the management of chronic diseases at the primary health care level in resource-poor setting.

Nurse-led care programs have been successfully implemented in Africa for chronic infectious diseases like HIV/AIDS [18, 19] tuberculosis [20] and to lesser extend leprosy [21], but little has been done for chronic NCD [22]. Our study therefore is of major contribution, by extending existing experience to NCDs. Our experience indicates that the program was more successful in the rural site, with regards to most outcomes. Many reasons can account for these findings: (1) compared with their urban counterparts, patients in the rural area had less opportunities of receiving care from physicians, unless they relocated in a nearby city. In the urban site, however, patients had a wide range of options to choose from, including receiving care from specialists. It would therefore appear that, once prompted on their condition during the survey or opportunistic screenings, urban patients will tend to report rather to physicians than nurses. This was likely the case for asthma and epilepsy, two conditions for which our urban clinics attracted no additional patients during the second year of the program. (2) The program was more subsidized in the rural area, at least for asthma and epilepsy with the provision of medications free of charge for the entire duration of the program. In a resource-limited setting this could be enough to increase clinic attendance among patients in the rural areas. (3) The rural population was more homogenous compared to the urban one, and nurses in charge of the rural clinics were members of the community. Patients therefore in the rural site could feel more secure with their usual care providers than urban participants. The most remarkable challenge in setting up primary care NCD clinics in urban areas is specialist shopping and specialised hospitals shopping by people with chronic NCD. However, when newly diagnosed patients settle to understand their chronic conditions, they come to terms with accepting primary care and going with it. However, a larger scale intervention in urban areas is required to build confidence in people with chronic NCD conditions.

Clinic forms developed within this programme were purposefully designed for use as stand alone. The build-in algorithms facilitate their use by any nurse with minimum basic training. Another advantage relates to the fact that follow-up information on consecutive visits for the same patients can stand on the same page. This will facilitate direct comparison of follow-up parameters, but also minimise the cost of production for follow-up forms. With up to twelve visits per year for example in our model, a patient with diabetes will need less than five double-side printed sheets, at the total cost of less than US$0.5 in Cameroon and many other African countries. Follow-up parameters included in our programme were limited to those easily obtainable at the primary health care level in most settings. Our models are therefore easily transposable to other African countries and we encourage our colleagues from other regions to adapt them as needed. It is also anticipated that in the future, computer user friendly of these tools will be developed to facilitate electronic data storage and information sharing among clinics.

This paper has not provided hard data to assess the performance of the clinics based on patients’ outcome on treatment, which is available in details elsewhere [14–17]. Although such information would have been of some contribution, their absence should not detract us from the fact that major step in the care of patients with chronic diseases resides in being able to drive them to health care facilities where any sustainable action can be initiated and monitored. Our intervention program was able to achieve this crucial step, and future action will build on this success to target patients’ outcome under treatment. Another limitation relates to the fact that we didn’t collect detailed information on screening activities, and are therefore unable to provide a response rate for patients referred from the baseline survey, but also to assess the diagnostic performances of nurses in the clinics.

## Conclusion

In the quest for cost-effective health care for NCD in SSA, rethinking health workforces and services delivery has some relevance. Nurse-led clinics, algorithm driven service delivery stands as alternatives to overcome the shortage of trained physicians and other issues relating to access to care. Because NCDs in this region are growing both in rural and urban areas, empowering more nurses has some potential for prevention and day-today management of chronic diseases. Nine years have elapsed since this program started and it has expanded into a national program for diabetes and hypertension. This program has also developed and made available a range of clinic tools easily adaptable in other resource-limited context.

## Figures and Tables

**Table 1:** Patients registered in clinics at the first and second year evaluations and percentage change

** Disease **	** Urban clinics **			** Rural clinics **		
	* 1st year *	* 2 ^ nd ^ year *	* Percent of change (95% CI) *	* 1 ^ st ^ year *	* 2 ^ nd ^ year *	* Percent of change (95% CI) *
Hypertension	115	120	4.3 (0.6–8)	159	234	47.2 (39.4–55)
Diabetes	38	180	373.7 (266.9–480.5)	28	38	35.7 (18–53.4)
Asthma	31	31	0	32	87	171.9 (133.4–210.4)
Epilepsy	11	11	0	135	224	65.9 (65.9–73.9)
** Total **	195	342	75.4 (69.4–81.4)	354	583	64.7 (59.7–69.7)

95% CI, 95% confidence interval

**Table 2:** Treatments received by patients at the last clinic evaluation in the two rural and three urban clinics

** Treatment **	** Rural public **	** Rural private **	** Urban 3 **	** Urban 1&2 **
*** Asthma ***				

Aminophilline	58.3%	100%	-	-
Salbutamol	41.7%	0%	-	-
Prednisolone	0%	0%	-	-
*** Epilepsy ***				

Phenobarbital	77.6%	92.2%	-	-
Phenitoin	19%	2.6%	-	-
Carbamazepine	0%	1.3%	-	-
Lifestyle modifications	3.4%	3.9%	-	-
*** Diabetes mellitus alone ***				

Lifestyle modifications	7.1%	0%	12.5%	9.5%
Glibenclamide	71.4%	33.3%	70.8%	23.8%
Gliclazide	0%	0%	2.1%	19.0%
Metformine	7.1%	33.3%	0%	4.8%
Metformine + Glibenclamide	14.4%	33.3%	2.1%	14.3%
Others	0%	0%	12.9%	28.6%
*** Diabetes mellitus with hypertension ***				

* Glucose lowering treatment *				
Lifestyle modifications	0%	0%	0%	9.1%
Glibenclamide	75.0%	100%	80.8%	36.4%
Gliclazide	0%	0%	0%	18.2%
Metformine	25.0%	0%	0%	4.5%
Metformine + Glibenclamide	0%	0%	2.1%	18.2%
Others	0%	0%	17.1%	13.6%
* Blood pressure lowering treatment *				
Lifestyle modifications	0%	0%	8.5%	4.5%
Hydrochlorothiazide (HCT)	33.3%	75%	42.5%	13.6%
Propranolol	16.7%	25%	0%	0%
Nifedipine	16.7%	0%	12.8%	13.6%
Methyldopa	0%	0%	6.4%	4.5%
Reserpine + Chlotalidone	0%	0%	0%	18.2%
Captopril	0%	0%	6.4%	13.6%
HCT + Propranolol	0%	0%	0%	4.5%
HCT + Nifedipine	33.3%	0%	17%	13.6%
HCT + Methyldopa	0%	0%	6.4%	4.5%
Others	0%	0%	0%	27.5%
*** Hypertension alone ***				

Lifestyle modifications	12.2%	8.7%	-	22.4%
HCT	30.6%	65.2%	-	22.4%
Propranolol	3.1%	1.4%	-	8.7%
Nifedipine	13.7%	10.1%	-	6.2%
Methyldopa	1.0%	4.3%	-	4.1%
Reserpine + Chlotalidone	1.0%	0%	-	10.2%
HCT + Captopril	0%	0%	-	2.0%
HCT + Propranolol	7.1%	0%	-	0%
HCT + Nifedipine	20.4%	5.8%	-	10.2%
HCT + Methyldopa	11.2%	4.3%	-	4.1%
Others	0%	0%	-	9.7%

Urban 1&2: 2 urban clinics started in the 1 ^st^ year; Urban 3: urban clinic started in the second year of the program for diabetic patients only. Urban 1 & 2 are collapsed to account for the significant two-ways movement of patients between the two clinics.

## Additional material

**
New patient forms** : http://www.panafrican-med-journal.com/content/article/3/10/annexes/annex1.pdf

**
Diabetes forms** : http://www.panafrican-med-journal.com/content/article/3/10/annexes/annex2.pdf

**
Hypertension follow-up and annual evaluation forms** : http://www.panafrican-med-journal.com/content/article/3/10/annexes/annex3.pdf

**
Asthma initial assessment and follow-up forms:** http://www.panafrican-med-journal.com/content/article/3/10/annexes/annex4.pdf

**
Epilepsy initial assessment and follow-up forms:** http://www.panafrican-med-journal.com/content/article/3/10/annexes/annex5.pdf
